# Supplementary material for: Spinal cord injury impairs cardiac function due to impaired bulbospinal sympathetic control
Source: Nat Commun. 2022 Mar 16;13:1382. doi: 10.1038/s41467-022-29066-1 (PMC8927412; doi:10.1038/s41467-022-29066-1)
Supplement: Supplementary file 1 — Supplementary Information [file 41467_2022_29066_MOESM1_ESM.pdf]

Spinal cord injury impairs cardiac function due to impaired bulbospinal sympathetic control

Fossey et al.

Supplementary Table 1. Participant characteristics of non-injured, sub-acute and chronic injured human groups

		GROUP		
	P VALUE	NON-INJURED	SUB-ACUTE	CHRONIC
Age (years)	p=0.674	39.6 (13.8)	42.7 (12.1)	41.2 (10.4)
Height (cm)	p=0.826	174 (8)	175 (11)	175 (10)
Weight (kg)	p=0.204	79.5 (12.2)	82.1 (15.4)	74.8 (12.3)
BSA (m^2)	p=0.141	1.94 (0.18)	1.98 (0.21)	1.90 (0.19)
Male/Female (% Male)	p=0.828	10/4 (71%)	14/9 (61%)	15/7 (68%)
AIS A/B (%A)	p=0.139	-	9/14 (39%)	14/8 (64%)
TSI (days)	**p<0.001**	-	95.6 (25.6)	5540.2 (3392.8)

Continuous values are means (SD). Non-injured (n = 14); Sub-acute SCI (n = 23): C3 (n = 1), C4 (n = 8), C5 (n = 7), C6 (n = 4), C7 (n = 1), C8 (n = 2); Chronic SCI (n = 22): C4 (n = 5), C5 (n = 8), C6 (n = 7), C7 (n = 2). Outliers were not removed prior to analysis as individuals were matched. Continuous variables were analyzed using either a one-way ANOVA and Tukey HSD post-hoc test (or Kruskal Wallis with post-hoc test, if non-parametric) or a Mann-Whitney U t-test for TSI (non-parametric). The categorical variables of sex and American Spinal Injury Association impairment scale (AIS) were analyzed using a Fisher's exact test. A, complete SCI; B, incomplete SCI; BSA, body surface area; TSI, time since injury. Bolded p values are significant, $p < 0.05$.

Supplementary Table 2. Temporal effects of SHAM and T3-SCI surgeries on pressure-volume data, hemodynamics and plasma norepinephrine in rodents

		GROUP					
	LR	SHAM	1 d	3 d	5 d	7 d	8 w
LV load-dependent systolic measures							
P_{max} (mmHg)	**p<0.001**	118.4 (3.8)	91.6 (9.6) ***	89.6 (13.0) ***	85.4 (8.5) ***	92.0 (5.8) ***	97.1 (8.5) ***
P_{es} (mmHg)	**p=0.014**	88.7 (8.5)	72.9 (9.5) *	76.2 (15.0)	66.8 (7.2) **	68.6 (16.5) **	80.5 (7.8)
P_{dev} (mmHg)	**p<0.001**	119.4 (4.6)	91.3 (5.8) ***	91.5 (14.3) ***	87.6 (8.4) ***	91.2 (3.4) ***	95.2 (8.1) ***
dP/dt_{max} (mmHg/s)	**p<0.001**	8383 (563)	5475 (846) ***	5240 (1825) ***	4935 (1156) ***	5765 (1178) ***	5597 (761) ***
SW (mmHg.mL)	**p<0.001**	20.4 (2.1)	18.6 (4.0)	14.4 (4.6) **	13.2 (3.0) **	14.4 (2.7) **	10.4 (4.7) ***
E_a (mmHg/µL)	**p=0.004**	0.43 (0.03)	0.32 (0.06)	0.45 (0.17)	0.39 (0.13)	0.36 (0.12)	0.69 (0.31)
LV load-independent systolic measures							
E_{es} (mmHg/µL)	**p<0.001**	2.81 (0.40)	0.85 (0.29) ***	1.29 (0.55) ***	1.10 (0.41) ***	1.10 (0.38) ***	1.50 (0.49) ***
E_a/E_{es}	**p=0.020**	0.16 (0.02)	0.36 (0.15) **	0.45 (0.38) **	0.36 (0.09) **	0.36 (0.16) **	0.39 (0.23) **
PRSW (mmHg)	p=0.484	121.1 (17.1)	121.4 (42.2)	121.1 (22.9)	106.4 (22.4)	141.3 (41.4)	138.6 (35.6)
LV load-dependent diastolic measures							
P_{ed} (mmHg)	p=0.620	4.49 (3.57)	5.03 (9.88)	2.12 (3.55)	1.47 (2.44)	3.19 (6.45)	3.68 (4.65)
$-dP/dt_{min}$ (mmHg/s)	**p<0.001**	-4957 (239)	-3217 (159) ***	-3008 (678) ***	-2750 (700) ***	-3514 (783) ***	-3190 (541) ***
tau (ms)	p=0.268	7.58 (0.85)	13.01 (7.29)	12.56 (5.77)	9.57 (1.12)	10.85 (3.95)	13.21 (6.85)
RC hemodynamic measures							
MAP (mmHg)	**p<0.001**	85.3 (9.9)	75.1 (7.0)	74.8 (11.7)	60.1 (7.2) ***	67.2 (7.4) **	69.2 (10.2) **
SBP (mmHg)	**p<0.001**	115.2 (7.9)	91.9 (3.9) ***	88.8 (11.1) ***	76.3 (8.6) ***	85.9 (5.5) ***	91.6 (8.2) ***
DBP (mmHg)	p=0.055	62.4 (10.3)	61.8 (7.3)	61.2 (10.1)	48.3 (6.1)	54.1 (6.7)	54.6 (11.4)
HR (bpm)	**p=0.014**	374 (29)	278 (40) **	276 (101) **	271 (59) **	334 (63)	276 (36) **
SVR (mmHg/mL)	p=0.123	1.11 (0.13)	1.22 (0.39)	1.69 (0.91)	1.46 (0.69)	1.16 (0.44)	1.89 (0.57)
Sympathetic activity measure							
Plasma NE (pg/mL)	**p=0.002**	2618 (312)	1145 (452) ***	-	-	1363 (693) **	1767 (470) *

Values are means (SD). For pressure-volume and hemodynamic data: SHAM (n = 6); 1 d: 1 day T3-SCI (n = 6-7); 3 d: 3 days T3-SCI (n = 5-6); 5 d: 5 days T3-SCI (n = 5-6); 7 d: 7 days T3-SCI (n = 6); 8 w: 8 weeks T3-SCI (n = 8-9). For NE data: SHAM (n = 4); 1 d (n = 7); 7 d (n = 6); 8 w (n = 5). Group differences for linear regression (LR) with coefficient (group mean differences) p-values are shown as symbols (SHAM *vs*. T3-SCI timepoints): *p < 0.05, **p < 0.01 and ***p < 0.001. If data were non-parametric, a Box-Cox transformation or a logarithmic transformation was performed. DBP, diastolic blood pressure; dP/dt_{max}, maximal rate of systolic pressure increment; $-dP/dt_{min}$, maximal rate of diastolic pressure decrement; E_a, arterial elastance; E_a/E_{es}, ventricular vascular coupling

ratio; E_{es}, end-systolic elastance (end-systolic pressure-volume relationship); HR, heart rate; LV, left ventricle; MAP, mean arterial pressure; NE, norepinephrine; P_{dev}, developed pressure; P_{ed}, end-diastolic pressure; P_{es}, end-systolic pressure; P_{max}, maximum pressure; PRSW, preload recruitable stroke work; RC, right-carotid; SBP, systolic blood pressure; SVR, systemic vascular resistance; SW, stroke work; tau, diastolic time constant. Bolded *p* values are significant, $p < 0.05$.

Supplementary Table 3. Temporal effects of SHAM and T3-SCI surgeries on gene expression, anatomical and cardiomyocyte dimensional data in rodents

		GROUP						
	LR	SHAM	12 h	1 d	3 d	5 d	7 d	8 w
RNA fold changes of UPS and Autophagy markers								
MAFbx (UPS)	**p<0.001**	1.06 (0.34)	3.57 (1.07) ***	2.33 (1.13) **	2.26 (0.42) **	1.93 (0.49) *	1.73 (0.48) *	-
MuRF1 (UPS)	p=0.271	1.02 (0.22)	1.38 (0.23)	1.38 (0.52)	1.49 (0.38)	1.66 (0.62)	1.03 (0.74)	-
BECN1 (Autophagy)	p=0.982	1.01 (0.13)	1.07 (0.33)	1.21 (0.54)	1.05 (0.23)	1.04 (0.31)	1.13 (0.24)	-
ATG7 (Autophagy)	p=0.355	1.03 (0.28)	1.64 (0.36)	1.36 (0.52)	1.30 (0.19)	1.32 (0.48)	1.59 (0.71)	-
ATG12 (Autophagy)	p=0.692	1.01 (0.19)	1.20 (0.28)	1.11 (0.35)	1.07 (0.24)	1.14 (0.45)	1.43 (0.55)	-
Anatomical measures at termination								
Body weight (g)	**p<0.001**	334 (8)	362 (42)	392 (52) *	343 (43)	333 (39)	315 (34)	401 (27) **
Femur length (cm)	**p<0.001**	3.54 (0.05)	3.51 (0.12)	3.63 (0.13)	3.53 (0.10)	3.57 (0.13)	3.58 (0.10)	4.07 (0.03) ***
Absolute LV cardiomyocyte dimensions								
Length (μm)	p=0.325	90.8 (6.1)	88.6 (4.9)	89.9 (5.7)	86.8 (7.2)	89.6 (6.8)	84.1 (5.8)	87.2 (2.1)
Width (μm)	p=0.789	15.7 (2.4)	14.8 (1.8)	14.2 (2.5)	14.1 (1.3)	14.8 (2.3)	14.5 (1.4)	14.9 (1.5)
CSA (μm^2)	p=0.056	465 (131)	488 (160)	330 (36)	446 (136)	400 (82)	353 (69)	350 (63)
Volume (μm^3)	p=0.114	42302 (13033)	43782 (15980)	30585 (1022)	38145 (13489)	34446 (6951)	29646 (5896)	30889 (4924)
Standardized LV cardiomyocyte dimensions								
Std length (μm/cm)	**p<0.001**	25.6 (2.0)	25.3 (2.0)	24.8 (1.5)	24.6 (1.9)	25.1 (2.5)	23.5 (1.8) *	21.5 (0.7) ***
Std width (μm/cm)	p=0.192	4.42 (0.70)	4.21 (0.54)	3.91 (0.64)	3.98 (0.39)	4.13 (0.61)	4.05 (0.42)	3.67 (0.36)
Std CSA ($\mu m^2/cm^2$)	**p<0.001**	37.0 (10.5)	40.0 (14.4)	25.2 (3.2) *	35.2 (9.0)	31.0 (8.2)	27.6 (5.7)	21.3 (3.7) ***
Std volume ($\mu m^3/cm^3$)	**p<0.001**	953 (303)	1033 (432)	623 (73) *	845 (228)	748 (220)	649 (143) *	462 (69) ***

Values are means (SD). For RNA fold changes: all groups (n = 5). For anatomical and histological measures: SHAM (n = 6); 12 h: 12 hours T3-SCI (n = 8); 1 d: 1 day T3-SCI (n = 6-7); 3 d: 3 days T3-SCI (n = 8 for all variables except CSA and volume measures, n = 5); 5 d: 5 days T3-SCI (n = 7 for all variables except CSA and volume measures, n = 5); 7 d: 7 days T3-SCI (n = 8); 8 w: 8 weeks T3-SCI (n = 8-9). Group differences for linear regression (LR) with coefficient (group mean differences) p-values are shown as symbols (SHAM *vs*. T3-SCI timepoints): *$p < 0.05$, **$p < 0.01$ and ***$p < 0.001$. If data were non-parametric, a Box-Cox transformation was performed. *ATG7*, autophagy related 7; *ATG12*, autophagy related 12; *BECN1*, beclin 1; CSA, cross-sectional area; *MAFbx*, muscle atrophy F-box; *MuRF1*, muscle RING-finger protein 1; LV, left ventricle; Std, standardized to femur length; UPS, ubiquitin proteasome system. Bolded p values are significant, $p < 0.05$.

Supplementary Table 4. qPCR primer sequences for protein degradation targets

Pathway	Target	Primer Sequence (forward and reverse)
UPS	*MAFbx*	5’-ACTTCTCAGAGCGGCAGATCC-3’
		5’-CTCTGGGTTGTTGGCCGT-3’
	MuRF1	5’-GCAGGAATGCTCCAGTCGG-3’
		5’-GTGAGCCCCGAACACCTT-3’
Autophagy	*BECN1*	5’-CGTCGGGGCCTAAAGAATG-3’
		5’-GCTCTCTCCTGGTTTCGCC-3’
	ATG12	5’-CCCAGAAACAGCCATCCCA-3’
		5’-TCACATAAATAAACAACTGCTCCGA-3’
	ATG7	5’-AGACCTTGAGCGTGCGTATG-3’
		5’-AACTGCTACTCCATCTGTGGG-3’
Housekeeping	*ACTB*	5’-GGGAAATCGTGCGTGACT-3’
		5’-GCGGCAGTGGCCATCTC-3’

ATG7, autophagy related 7; *ATG12*, autophagy related 12; *ACTB*, $\boldsymbol{\beta}$-actin; *BECN1*, beclin 1; *MAFbx*, muscle atrophy F-box; *MuRF1*, muscle RING-finger protein 1; UPS, ubiquitin proteasome system.

Supplementary Table 5. Effects of T3-SCI and combined T3-SCI with hexamethonium bromide, ganglionic blockader, on pressure-volume data in rodents

		CONDITION		
	RM-ANOVA	Pre-intervention	Post-SCI	Post-HEX
LV load-dependent systolic measures				
P_{max} (mmHg)	**p<0.001**	113.5 (7.9)	70.1 (7.3) ***	58.0 (15.6) ***
P_{es} (mmHg)	**p<0.001**	91.2 (9.6)	54.0 (7.4) ***	51.0 (15.3) **
P_{dev} (mmHg)	**p<0.001**	115.8 (6.4)	72.6 (7.5) ***	59.1 (16.1) ***
dP/dt_{max} (mmHg/s)	**p<0.001**	7313 (880)	3421 (858) ***	2094 (746) ***
SW (mmHg.mL)	**p<0.001**	30.61 (13.04)	14.75 (6.87) **	13.00 (6.81) **
E_a (mmHg/µL)	p=0.099	0.34 (0.10)	0.28 (0.09)	0.22 (0.11)
LV load-dependent diastolic measures				
P_{ed} (mmHg)	p=0.176	1.88 (2.71)	-0.06 (1.60)	0.90 (1.56)
$-dP/dt_{min}$ (mmHg/s)	**p<0.001**	-4767 (735)	-2365 (586) ***	-1677 (950) *** †
tau (ms)	**p=0.003**	64.7 (34.1)	72.1 (37.9)	170.3 (88.9) *
LV volumetric measures				
EDV (µL)	**p=0.015**	274 (116)	258 (122)	403 (156)
ESV (µL)	**p=0.005**	70.6 (39.4)	75.9 (46.5)	186.3 (95.3) *
SV (µL)	p=0.110	297 (110)	215 (74)	260 (83)
Q (mL/min)	p=0.026	103.9 (53.2)	69.5 (36.1) *	69.4 (25.7)
HR (bpm)	**p<0.001**	335 (61)	307 (76)	262 (55) ***

Values are means (SD) with $n = 7$. Differences between conditions for one-way repeated-measures (RM)-ANOVA with paired t-test post-hoc (Bonferroni correction) are shown with symbols. Comparison *vs.* pre-intervention: *p < 0.05, **p < 0.01 and ***p < 0.001; comparison *vs.* post-SCI: : †p < 0.05, ††p < 0.01 and †††p < 0.001. dP/dt_{max}, maximal rate of systolic pressure increment; $-dP/dt_{min}$, maximal rate of diastolic pressure decrement; E_a, arterial elastance; EDV, end-diastolic volume; ESV, end-systolic volume; HEX, hexamethonium bromide; HR, heart rate; LV, left ventricle; P_{dev}, developed pressure; P_{ed}, end-diastolic pressure; P_{es}, end-systolic pressure; P_{max}, maximum pressure; Q, cardiac output; SV, stroke volume; SW, stroke work; tau, diastolic time constant. Bolded *p* values are significant, $p < 0.05$.

Supplementary Table 6. Effects of intact (L2-SCI) and reduced (T3-SCI) descending sympathetic control on pressure-volume data and hemodynamics in rodents

			GROUP	
	ANOVA	SHAM	T3-SCI	L2-SCI
LV load-dependent systolic measures				
P_{max} (mmHg)	**p=0.002**	116.8 (14.7)	82.7 (9.2) **	121.0 (10.0) ††
P_{es} (mmHg)	**p=0.002**	82.5 (9.3)	63.2 (9.0) *	90.5 (14.0) ††
P_{dev} (mmHg)	**p<0.001**	116.2 (14.1)	81.1 (11.4) ***	117.9 (6.3) †††
dP/dt_{max} (mmHg/s)	**p<0.001**	11424 (1664)	7015 (1538) **	13586 (2136) †††
SW (mmHg.mL)	**p<0.001**	22.30 (6.96)	8.50 (2.69) **	22.47 (6.10) ††
E_a (mmHg/μL)	p=0.458	0.40 (0.15)	0.48 (0.12)	0.40 (0.08)
LV load-independent systolic measures				
E_{es} (mmHg/μL)	**p=0.042**	1.35 (0.24)	0.90 (0.26)	1.37 (0.46)
E_a/E_{es}	**p=0.015**	0.30 (0.11)	0.56 (0.15) **	0.32 (0.13) †
PRSW (mmHg)	**p=0.005**	130.0 (16.4)	71.2 (30.5) **	104.8 (29.4)
LV load-dependent diastolic measures				
P_{ed} (mmHg)	p=0.964	5.74 (1.87)	5.33 (1.91)	6.04 (2.57)
$-dP/dt_{min}$ (mmHg/s)	**p<0.001**	-6463 (677)	-4191 (953) **	-7169 (1479) †††
tau (ms)	p=0.132	6.83 (1.00)	7.64 (1.89)	8.84 (1.99)
RC hemodynamic measures				
MAP (mmHg)	**p<0.001**	90.0 (11.9)	51.7 (5.8) ***	87.4 (12.9) †††
SBP (mmHg)	**p=0.002**	113.4 (12.1)	71.2 (7.5) **	115.9 (3.3) ††
DBP (mmHg)	**p<0.001**	75.2 (12.4)	42.7 (5.6) ***	70.1 (14.9) ††
HR (bpm)	p=0.779	388 (38)	347 (147)	384 (28)
SVR (mmHg/mL)	p=0.919	0.97 (0.25)	0.94 (0.20)	1.00 (0.29)
LV volumetric measures				
EDV (μL)	**p=0.015**	261 (77)	143 (17) *	251 (71) ††
ESV (μL)	p=0.055	49.9 (24.1)	20.3 (9.0)	32.6 (23.2)
SV (μL)	**p=0.005**	240 (68)	137 (25) **	235 (55) †
Q (mL/min)	**p<0.001**	96.5 (19.6)	56.3 (7.8) **	91.8 (18.9) ††

Values are means (SD). SHAM (*n* = 6-7); T3-SCI (*n* = 6); L2-SCI (*n* = 6-7). Group differences for one-way ANOVA with Tukey HSD post-hoc (or Kruskal Wallis with Mann-Whitney U post-hoc tests, if non-parametric) are shown with symbols. Between-group comparison *vs.* SHAM: *$p < 0.05$, **$p < 0.01$ and ***$p < 0.001$; between-group comparison *vs.* T3-SCI: †$p < 0.05$, ††$p < 0.01$ and †††$p < 0.001$. DBP, diastolic blood pressure; dP/dt_{max}, maximal rate of systolic pressure increment; $-dP/dt_{min}$, maximal rate of diastolic pressure decrement; E_a, arterial elastance; E_a/E_{es}, ventricular vascular coupling ratio; EDV, end-diastolic volume; E_{es}, end-systolic elastance (end-systolic pressure-volume relationship); ESV, end-systolic volume; HR, heart rate; LV, left ventricle; MAP, mean arterial pressure; P_{dev}, developed pressure; P_{ed}, end-diastolic pressure; P_{es}, end-systolic pressure; P_{max}, maximum pressure; PRSW, preload recruitable stroke work; Q, cardiac output; RC, right-carotid; SBP, systolic blood pressure; SV, stroke volume; SVR, systemic vascular resistance; SW, stroke work; tau, diastolic time constant. Bolded *p* values are significant, $p < 0.05$.

Supplementary Table 7. Effects of minocycline on pressure-volume data and hemodynamics following severe T3 contusion in rodents

	TS T-TEST	GROUP	
		Vehicle	Minocycline
LV load-dependent systolic measures			
Pmax (mmHg)	**p=0.009**	95.8 (13.7)	123.1 (13.2)
Pes (mmHg)	**p=0.005**	81.0 (13.5)	109.8 (12.1)
Pdev (mmHg)	p=0.073	86.5 (8.3)	97.0 (9.0)
dP/dtmax (mmHg/s)	p=0.089	5470 (2120)	8406 (2989)
SW (mmHg.mL)	p=0.662	7.58 (3.96)	8.27 (3.92)
Ea (mmHg/µL)	p=0.090	0.81 (0.27)	1.25 (0.48)
LV load-independent systolic measures			
E_{es} (mmHg/µL)	**p=0.022**	0.71 (0.33)	1.38 (0.46)
E_a/E_{es}	p=0.931	1.32 (0.63)	1.79 (1.95)
PRSW (mmHg)	p=0.301	69.8 (23.7)	89.8 (36.6)
LV load-dependent diastolic measures			
P_{ed} (mmHg)	**p=0.023**	13.3 (9.5)	26.1 (4.5)
$-dP/dt_{min}$ (mmHg/s)	**p=0.001**	-4692 (680)	-6938 (972)
tau (ms)	p=0.247	15.3 (7.7)	21.0 (6.0)
RC hemodynamic measures			
MAP (mmHg)	p=0.185	81.4 (8.8)	88.3 (6.7)
SBP (mmHg)	p=0.487	101.3 (8.7)	104.7 (6.3)
DBP (mmHg)	p=0.105	71.4 (8.9)	80.3 (7.0)
HR (bpm)	p=0.910	325 (63)	320 (61)
SVR (mmHg/mL)	p=0.956	3.07 (1.29)	3.11 (1.19)
LV volumetric measures			
EDV (µL)	p=0.488	149 (42)	175 (75)
ESV (µL)	p=0.425	66.9 (23.7)	85.7 (49.0)
SV (µL)	p=0.666	110 (42)	99 (38)
Q (mL/min)	p=0.931	31.1 (15.0)	33.0 (15.7)
Histology - RVLM neuronal counts			
DBH+ neurons	-	389 (72)	319 (144)
FG+ neurons	-	129 (210)	368 (235)
DBH+/FG+/NeuN+ neurons	-	30 (52)	63 (39)

Values are means (SD). Catheter-derived measures: Vehicle (*n* = 6); Minocycline (*n* = 5). Histological counts: all groups (*n* = 4). Group differences were analyzed using a two-sample (TS) t-test (or Mann-Whitney U test, if non-parametric). DBH, dopamine beta-hydroxylase; DBP, diastolic blood pressure; dP/dt_{max}, maximal rate of systolic pressure increment; $-dP/dt_{min}$, maximal rate of diastolic pressure decrement; Ea, arterial elastance; E_a/E_{es}, ventricular vascular coupling ratio; EDV, end-diastolic volume; E_{es}, end-systolic elastance (end-systolic pressure-volume relationship); ESV, end-systolic volume; FG, FluoroGold; HR, heart rate; LV, left ventricle; MAP, mean arterial pressure; NeuN, neuronal nuclei; P_{dev}, developed pressure; P_{ed}, end-diastolic pressure; P_{es}, end-systolic pressure;

P_{max}, maximum pressure; PRSW, preload recruitable stroke work; Q, cardiac output; RC, right-carotid; RVLM, rostral ventrolateral medulla; SBP, systolic blood pressure; SV, stroke volume; SVR, systemic vascular resistance; SW, stroke work; tau, diastolic time constant; +, positive. Bolded *p* values are significant, $p < 0.05$.

Supplementary Table 8. Effects of penile vibrostimulation on hemodynamics, heart rate and systolic function measures in men with cervical SCI

		CONDITION	
	TS t-test	Pre-intervention	PVS
Hemodynamics and heart rate measures			
MAP (mmHg)	**p<0.001**	67.5 (7.0)	97.4 (11.0)
SBP (mmHg)	**p<0.001**	93.2 (12.5)	143.7 (24.3)
DBP (mmHg)	**p<0.001**	54.7 (5.70)	74.3 (6.98)
HR (bpm)	**p=0.003**	69.4 (11.8)	55.8 (7.8)
Systolic function measures			
SV (mL)	**p=0.005**	59.0 (13.4)	70.1 (13.7)
Q (L/min)	p=0.346	3.99 (0.79)	3.78 (0.66)
dP/dt_{max} (mmHg/s)	**p=0.001**	498 (167)	856 (256)

Values are means (SD) with a total of n = 10 cervical SCI participants (AIS A, n = 5; AIS B, n = 5). Differences between pre-intervention and during penile vibrostimulation (PVS) were analyzed using a two-sample paired (TS) t-test. DBP, diastolic blood pressure; dP/dt_{max}, maximal rate of systolic pressure increment; HR, heart rate; MAP, mean arterial pressure; Q, cardiac output; SBP, systolic blood pressure; SV, stroke volume. Bolded p values are significant, $p < 0.05$.
