# Supplementary material for: Spinal cord injury impairs cardiac function due to impaired bulbospinal sympathetic control
Source: Nat Commun. 2022 Mar 16;13:1382. doi: 10.1038/s41467-022-29066-1 (PMC8927412; doi:10.1038/s41467-022-29066-1)
Supplement: Supplementary file 3 — Description of additional supplementary files [file 41467_2022_29066_MOESM3_ESM.docx]

**Description of additional supplementary information:**

**Supplementary Data 1 Title: Echocardiographic measure differences between non-injured, sub-acute and chronic injured human groups**

**Supplementary Data 1** **Legend:** Values are means (SD). Volumetric, functional and structural measures: non-injured (n = 12-14); sub-acute (n = 13-23); chronic (n = 8-22). Mechanical and strain measures: non-injured (n = 11-14); sub-acute (n = 10-15); chronic (n = 12-19). Group differences for one-way non-repeated measures ANOVA with Tukey HSD post-hoc (or Kruskal Wallis with Mann-Whitney U post-hoc, if non-parametric) are shown with symbols. Between-group comparison (vs. non-injured): *p < 0.05, **p < 0.01 and ***p < 0.001; (vs. sub-acute): †p < 0.05, ††p < 0.01 and †††p < 0.001. A, late transmitral filling velocity; d, end-diastolic; E, early transmitral filling velocity; E’, early transmitral myocardial velocity; EDV, end-diastolic volume; EF, ejection fraction; ESV, end-systolic volume; HR, heart rate; IVRT, isovolumetric relaxation time; IVS, intraventricular septum thickness; LV, left ventricle; LVID, left ventricular internal diameter; PW, posterior wall thickness; Q, cardiac output; RWT, relative wall thickness; s, end-systolic; S’, myocardial contractile velocity; SV, stroke volume. Bolded p values are significant, p < 0.05.

**Supplementary Data 2 Title: Echocardiography-derived outcomes following SHAM and T3-SCI surgeries over the acute setting in rodents**

**Supplementary Data 2** **Legend:** Values are means (SD). All variables except MV E: SHAM (n = 6); T3-SCI (n = 6-8). MV E: SHAM (n = 5); T3-SCI (n = 3). Effects from two-way repeated measures (RM)-ANOVA are shown for time, group and interaction (group x time). Two-sample (TS) t-test (or Mann-Whitney U test, if non-parametric) was performed post-hoc for between-group comparisons, and one-way RM-ANOVA with Bonferroni corrected pairwise dependent samples t-test (or Wilcoxon signed-rank test, if non-parametric) for within-group comparisons. Between-group comparison (SHAM vs. T3-SCI): *p < 0.05, **p < 0.01 and ***p < 0.001. Within-group comparison to control (i.e., pre-surgery for absolute values and 1 d post-surgery for percent change (%∆)): †p < 0.05, ††p < 0.01 and †††p < 0.001. Between-time comparison to control for non-significant interactions (i.e., pre-surgery for absolute values and 1 d post-surgery for %∆): ‡‡‡ p < 0.001. d, end-diastolic; EDV, end-diastolic volume; EF, ejection fraction; ESV, end-systolic volume; HR, heart rate; IVS, intraventricular septum thickness; LV, left ventricle; LVID, left ventricular internal diameter; LVPW, left ventricular posterior wall thickness; MV E, mitral valve E velocity; Q, cardiac output; RWT, relative wall thickness; s, end-systolic; SV, stroke volume. Bolded p values are significant, p < 0.05.

**Supplementary Data 3 Title: Echocardiography-derived outcomes following SHAM and T3-SCI surgeries over the chronic setting in rodents**

**Supplementary Data 3** **Legend:** Values are means (SD). SHAM (n = 6-7); T3-SCI (n = 7-10). For absolute value comparisons (including pre-surgery), effects from two-way repeated measures (RM)-ANOVA are shown for time, group and interaction (group x time) with two-sample (TS) t-test (or Mann-Whitney U test, if non-parametric) was performed post-hoc for between-group comparisons and for within-group comparisons. For percent change (%∆), TS t-test (or Mann-Whitney U test, if non-parametric) was performed. Between-group comparison (SHAM vs. T3-SCI): *p < 0.05, **p < 0.01 and ***p < 0.001. Within-group comparison to pre-surgery: †p < 0.05 and ††p < 0.01. See Supplementary Table 2a for abbreviations. Bolded p values are significant, p < 0.05.
