# Supplementary material for: Spinal cord injury impairs cardiac function due to impaired bulbospinal sympathetic control
Source: Nat Commun. 2022 Mar 16;13:1382. doi: 10.1038/s41467-022-29066-1 (PMC8927412; doi:10.1038/s41467-022-29066-1)
Supplement: Supplementary file 7 — Reporting Summary [file 41467_2022_29066_MOESM7_ESM.pdf]

nature research

Corresponding author(s): Dr. Christopher R. West
Dr. Andrei V. Krassioukov

Last updated by author(s): Feb 10, 2022

Reporting Summary

Nature Research wishes to improve the reproducibility of the work that we publish. This form provides structure for consistency and transparency in reporting. For further information on Nature Research policies, see our Editorial Policies and the Editorial Policy Checklist.

Statistics

For all statistical analyses, confirm that the following items are present in the figure legend, table legend, main text, or Methods section.

n/a	Confirmed	
☐	☒	The exact sample size (*n*) for each experimental group/condition, given as a discrete number and unit of measurement
☐	☒	A statement on whether measurements were taken from distinct samples or whether the same sample was measured repeatedly
☐	☒	The statistical test(s) used AND whether they are one- or two-sided<br>*Only common tests should be described solely by name; describe more complex techniques in the Methods section.*
☒	☐	A description of all covariates tested
☐	☒	A description of any assumptions or corrections, such as tests of normality and adjustment for multiple comparisons
☐	☒	A full description of the statistical parameters including central tendency (e.g. means) or other basic estimates (e.g. regression coefficient) AND variation (e.g. standard deviation) or associated estimates of uncertainty (e.g. confidence intervals)
☐	☒	For null hypothesis testing, the test statistic (e.g. *F*, *t*, *r*) with confidence intervals, effect sizes, degrees of freedom and *P* value noted<br>*Give P values as exact values whenever suitable.*
☒	☐	For Bayesian analysis, information on the choice of priors and Markov chain Monte Carlo settings
☒	☐	For hierarchical and complex designs, identification of the appropriate level for tests and full reporting of outcomes
☒	☐	Estimates of effect sizes (e.g. Cohen's *d*, Pearson's *r*), indicating how they were calculated

Our web collection on statistics for biologists contains articles on many of the points above.

Software and code

Policy information about availability of computer code

Data collection	Human data collected in Part I: Echocardiography data was acquired via a Vivid 7 / i ultrasound unit (GE Healthcare, Mississauga, ON).<br>Human data collected in Part III: Cardiovascular measures were acquired via a finometer (Finapres Medical Systems BV, Arnhem, The Netherlands) and a standard 3-lead electrocardiogram (lead II; Powerlab Model ML132). Penile vibrostimulation was performed via a WAHL (WAHL model 4196, Div Swenson Canada Inc, Toronto, ON, Canada) or Ferticare Clinic vibrator (Multicept APS, Rungsted, Denmark).<br><br>Animal data collected in Part I and II: Echocardiography data were acquired via a high-frequency animal ultrasound machine (Vevo 3100; FUJIFILM VisualSonics, Toronto, ON, Canada) and a high-frequency cardiac transducer (MS250, 13-24 MHz; VisualSonics, Toronto, ON, Canada). Raw data from the left ventricular (LV) pressure-volume catheter were acquired via a pressure-volume catheter (1.9F rat PV catheter with an ADV500 PV system, Transonic®, Ithaca, NY, USA; or, SPR-869; Millar, Inc., Houston, TX, USA). All LV pressure-volume data, cardiac output, blood pressure and heart rate were monitored and acquired using LabChart version 8.1 (PowerLab16/35; ADInstruments, Colorado Springs, CO, USA). Measures of cardiac output, as well as LV load-dependent and load-independent variables were made using the LabChart PV Loop Analysis and Cardiac Output modules. Norepinephrine data was measured via enzyme linked immunoabsorbent assay (ELISA; BA E-5200R Norepinephrine Research ELISA; Labor Diagnostika, Nordhorn, Germany). ELISA optical densities were measured with the iMark microplate reader at 450 nm as per manufacturer instruction (BioRad, CA, USA) with Microplate Manager® Software version 6.3 (MPM6, BioRad). ELISA standard curves and concentrations were automatically calculated using MPM6 software using a four-parameter fit. Gene expression data was acquired via the NanoDropTM 2000 spectrophotometer (Thermo Fisher Scientific Inc., Wilmington, DE, USA) for RNA purity and via the Applied BiosystemsTM ViiA 7 Real-Time PCR System (Life Technologies Corporation, Thermo Fisher Scientific Inc., Carlsbad, CA, USA) for cDNA quantification. Histology data were acquired via a confocal microscope (ZEISS Axio Observer and Yokogawa Spinning Disk; ZEISS, Oberkochen, Germany).
Data analysis	Human data analysis in Part I: Echocardiography data was analyzed via EchoPAC (version 202; GE Healthcare, Horten, Norway) and a speckle-training software (2D Strain Analysis Tool, Stuttgart, Germany).<br>Human data analysis in Part III: Penile vibrostimulation data was stored and analyzed via LabChart (described above).

Animal data analysis in Part I and II: Echocardiography data were analyzed via Vevo LAB software (VisualSonics, Toronto, ON, Canada). LV pressure-volume data were analyzed via LabChart (described above). Histological data were analyzed via Fiji ImageJ (version 1.52e).

All statistical analyses were performed using R Studio (version 1.3.959, R Studio Team, PBC, Boston, MA) and R (4.0.1 GUI 1.72 Catalina build 7845, R Foundation for Statistical Computing, R Core Team, Vienna, Austria).

Graphical representations were made in Prism (version 6.0e, GraphPad Software, San Diego, CA, USA) and Adobe Illustrator (version 13.1.1, Adobe Inc., 2019, San Jose, CA, USA)

For manuscripts utilizing custom algorithms or software that are central to the research but not yet described in published literature, software must be made available to editors and reviewers. We strongly encourage code deposition in a community repository (e.g. GitHub). See the Nature Research guidelines for submitting code & software for further information.

Data

Policy information about availability of data

All manuscripts must include a data availability statement. This statement should provide the following information, where applicable:

- Accession codes, unique identifiers, or web links for publicly available datasets
- A list of figures that have associated raw data
- A description of any restrictions on data availability

Source data for figures 2-9 are provided as a Source Data Files.

Field-specific reporting

Please select the one below that is the best fit for your research. If you are not sure, read the appropriate sections before making your selection.

☒ Life sciences ☐ Behavioural & social sciences ☐ Ecological, evolutionary & environmental sciences

For a reference copy of the document with all sections, see nature.com/documents/nr-reporting-summary-flat.pdf

Life sciences study design

All studies must disclose on these points even when the disclosure is negative.

Sample size

Human participant sample size: Using previously published data from our group with end-diastolic volume as a measure (doi: 10.1089/neu.2016.4510), we determined that a sample size of nine human participants per group was sufficient for 80% power, an α of 0.05 and an effect size of 1.45 to detect a mean difference of 27 mL and SD of 5 mL between non-injured and untrained tetraplegic individuals (G*Power, version 3.1.9.7).

Animal sample size: Using previous data for our key outcome (end-systolic elastance Ees) from our research group (doi: 10.1089/neu.2017.5624), we determined that a sample size of four rats per group for pressure volume data was sufficient for 80% power, an α of 0.05 and an effect size of 1 to detect a significant mean difference of 0.55 mmHg µL-1 with a pooled SD of 0.25 mmHg µL-1 between rats with a T3-SCI and SHAM injury (G*Power).

Data exclusions

Human participants in Part I: A total of 59 human echocardiography studies were analyzed. Delineating the endocardial borders can be a limitation in measuring the area of the left ventricle in certain views. All measures were attempted for all participants, with specific group sizes provided in the manuscript.
Human participants in Part III: Ten participants were selected to present a dramatic rise in blood pressure during a common clinical procedure. As such, a selected time frame during the procedure to highlight the differences were presented, as shown in the figure. No exclusions were performed in this study.

Animals in Part I: A total of 66 animals were initially allocated to groups. However, four animals (6.1%) died during or immediately following the high-thoracic spinal cord injury surgery. A death rate of 10% for spinal cord injured animals is deemed by the University of British Columbia Animal Care Committee. Additionally, one animal was excluded due to poor health at termination. Although we attempted to conduct all outcome assessments in every animal, we were unable to catheterize some animals, whilst others had poor perfusion of tissue. The specific sample sizes per group are provided in the manuscript.
Animals in Part II: No animals were excluded.

Replication

We did not attempt to replicate our experimental findings However, since our data we collected in individual studies by extension we have demonstrated that the reduction in pressure and contractility indices with spinal cord injury is consistent across studies. In our histology analyses we ensured we measured cardiomyocyte length, width and cross sectional area from at least 98 cardiomyocytes (A minimum of 130 lengths, 210 widths and 98 CSAs) and then graphically represented these in a histogram. In this way we can be more certain that SCI causes cardiomyocyte atrophy then just representing the mean of all measures per animal.

Randomization

In the human studies, human participants were not randomized as group identification was based on the presence/absence of spinal cord injury and the time since spinal cord injury. In the animal studies, all animals were randomized into groups.

Blinding

Humans in Part I: Blinding was not possible for the sonographer acquiring the data as location of participants (i.e., community dwelling or in

rehabilitation) and mobility of non-injured individuals could visually be distinguished. However, echocardiography analysis was blinded.
Humans in Part III: We were not blinded as there was only ten individuals of the same group (i.e., chronic cervical spinal cord injury) involved. These individuals presented to the clinic for sperm retrieval for personal incentives and consented for data to be collected and used for research purposes.

Animals in Part I: Blinding during surgeries and animal care was not possible for the surgeon and care staff as spinal cord injury leads to visual differences (i.e., hindlimb function and atrophy). However, all echocardiography, pressure-volume, ELISA, gene expression and histological analyses the individual was blinded to the animal's group.
Animals in Part II: For the ganglionic blockade and level of injury studies, blinding was not possible during surgeries and animal care as described above. For the minocycline study, blinding was possible during treatment administration as groups had the same severity and level of spinal cord injury. All pressure-volume and histological analyses the individual was blinded to the animal's group/treatment.

Reporting for specific materials, systems and methods

We require information from authors about some types of materials, experimental systems and methods used in many studies. Here, indicate whether each material, system or method listed is relevant to your study. If you are not sure if a list item applies to your research, read the appropriate section before selecting a response.

Materials & experimental systems

n/a	Involved in the study
☐	☒ Antibodies
☒	☐ Eukaryotic cell lines
☒	☐ Palaeontology and archaeology
☐	☒ Animals and other organisms
☐	☒ Human research participants
☒	☐ Clinical data
☒	☐ Dual use research of concern

Methods

n/a	Involved in the study
☒	☐ ChIP-seq
☒	☐ Flow cytometry
☒	☐ MRI-based neuroimaging

Antibodies

Antibodies used

Heart histology:
1) Wheat germ agglutinin (WGA) Alexa Fluor® 488 conjugate (1:2000, W11261; Life Technologies Corporation, Thermo Fisher Scientific Inc., Eugene, OR, USA);
2) Alpha-actinin (rabbit primary 1:400, EP2529Y, Abcam, Cambridge, MA, USA; donkey anti-rabbit secondary Alexa Fluor® 546 1:1000, 711-586-152, Jackson ImmunoResearch, West Grove, PA, USA);
3) Connexin-43 (goat primary 1:1000, NBP1-51938, Novus Biologicals, Oakville, ON, Canada; donkey anti-goat Alexa Fluor® 647 secondary 1:1000, 705-606-147, Jackson ImmunoResearch, West Grove, PA, USA);
4) Nuclei/DNA (Hoechst 33342 1:10000, H3570, Thermo Fischer Scientific Inc., Eugene, OR, USA).

Rostral ventrolateral medulla histology:
1) Neuronal nuclei (NeuN; guinea pig primary 1:500, ABN90P, MilliporeSigma, Burlington, MA, USA; donkey anti-guinea pig secondary Alexa Fluor® 647 1:200, 706-606-148, Jackson ImmunoResearch, West Grove, PA, USA);
2) Dopamine beta-hydroxylase (DBH; mouse primary 1:400, MAB308, MilliporeSigma, Burlington, MA, USA; donkey anti-mouse secondary Alexa Fluor® 488 1:200, 715-546-151, Jackson ImmunoResearch, West Grove, PA, USA).

Validation

Heart histology:
1) WGA: We used the manufacturer's suggestion to diluting the 1.0 mg/mL into Hank's balanced salt solution then using 5.0 ug/mL for pre-fixed mammalian cells (1:200). This product has not been utilized to stain rat cardiomyocytes but has been used to stain immature human induced pluripotent stem cells derived from cardiomyocytes (1:1000, doi: 10.1038/s41596-019-0189-8) and human pluripotent stem cell derived ventricular progenitors transplanted into mice (1:200, doi: 10.1016/j.ymthe.2018.02.012). After troubleshooting different dilutions, we found that 1:2000 was the optimal dilution as more concentrated lead to high background.
2) Alpha-actinin: This primary antibody has been used twice in our laboratory to stain alpha-actinin bands in rat cardiomyocytes (1:100, doi: 10.1089/neu.2017.4984; 1:100, doi: 10.1089/neu.2017.5624). Our current dilution differs slightly as the past dilutions were optimized for frozen tissue, whilst our current dilution (1:400) was optimized for paraffin-embedded tissue.
3) Connexin-43: This primary antibody has been used twice in our laboratory to stain connexin-43 stained intercalated discs in rat cardiomyocytes (1:250, doi: 10.1089/neu.2017.4984; 1:250, doi: 10.1089/neu.2017.5624). As above, our current dilution (1:1000) differs slightly as our current dilution was optimized for paraffin-embedded tissue.
4) Nuclei/DNA: This antibody has been used twice in our laboratory to stain nuclei in rat cardiomyocytes (1:1000, doi: 10.1089/neu.2017.4984; 1:5000, doi: 10.1089/neu.2017.5624). As above, our current dilution (1:10000) differs slightly as our current dilution was optimized for paraffin-embedded tissue.

Rostral ventrolateral medulla histology:
1) NeuN: The manufacturer indicates that this antibody reacts with both mouse and rat tissue and suggests a 1:1000 dilution for mouse frontal cortex tissue. This antibody has been utilized to stain rat neurons in frozen tissue (doi: 10.1371/journal.pone.0105752). After troubleshooting different dilutions, we found that 1:500 was the optimal dilution for frozen tissue. The same protocol has been followed by our research group (doi: 10.1089/neu.2018.5703).
2) DBH: The manufacturer suggests a 1:300-500 dilution for rat brain tissue. This antibody has been utilized to stain rat catecholaminergic neurons in frozen brain tissue (1:500, doi: 10.1152/ajpregu.00542.2012; 1:500, doi: 10.1371/

journal.pone.0062410). After troubleshooting different dilutions, we found that 1:400 was the optimal dilution for frozen tissue. The same protocol has been followed by our research group (doi: 10.1089/neu.2018.5703).

Note that for all histology positive and negative control were performed to confirm specificity.

Animals and other organisms

Policy information about studies involving animals; ARRIVE guidelines recommended for reporting animal research

Laboratory animals	Animals in Part I: 66 male Wistar rats aged 10-11 weeks (300-400 g; Envigo, USA) were acquired.<br>Animals in Part II: For the ganglionic blokade study (hexamethonium), four male Sprague Dawley rats aged 23-32 weeks (440-475 g; Envigo, USA) and three Wistar rats aged 11-12 weeks (350-450 g; Charles River, Canada) were acquired. For the level of injury study, 20 male Wistar rats aged 10-11 weeks (300-400 g; Envigo, USA) were acquired. For the minocycline study, 11 male Wistar rats aged 10-12 weeks (250-350 g; Charles River, Canada) were acquired.<br>All animals were housed in the ICORD vivarium for 1-2 weeks prior to surgery.
Wild animals	The study did not involve wild animals.
Field-collected samples	The study did not involve field-collected samples.
Ethics oversight	All protocols and procedures were compliant with Canadian Council on Animal Care policies, and ethical approval was obtained from the University of British Columbia Animal Care Committee (A18-0344 and A14-0152).

Note that full information on the approval of the study protocol must also be provided in the manuscript.

Human research participants

Policy information about studies involving human research participants

Population characteristics	Human participants in Part I: 45 individuals with spinal cord injury between the ages of 18 and 60 years old, both male and female, with no history of cardiac disease, able to understand English, and had no history of cognitive impairments (able to follow instructions and no history of drug abuse). Of these 45 individuals, 23 had sub-acute spinal cord injury and 22 has chronic spinal cord injury. We recruited 14 non-injured controls. Groups were age and sex matched.<br>Human participants in Part III: Ten male individuals with chronic cervical spinal cord injury. These individuals presented to the clinic for sperm retrieval for personal incentives and consented for data to be collected and used for research purposes.
Recruitment	Non-injured controls (n=14, n=11 males, age range: 22-63yrs) were recruited from the community via the BSCC from February 2017-April 2018 and were comparable to both age and sex to SCI individuals. Exclusion criteria comprised any history of cardiovascular disease, which was confirmed with a verbal medical history, and any language or cognitive barrier that prevented the individual from following English instructions. The purpose for the recruitment of this group was to provide comparable demographics to the SCI groups. Though the group is taken from a convenience sample, it likely represents the larger population of non-injured individuals living in an urban and developed country that fit the inclusion and exclusion criteria of the study.<br><br>Individuals in the sub-acute group (n=23, n=14 males, age range: 28-60yrs) were consecutively recruited from the GF Strong Rehabilitation Centre, Vancouver, BC, Canada from April 2014-December 2018. These individuals were recruited following admission to the rehabilitation centre and provided informed consent to participate in the study. These individuals were then screened to meet the inclusion and exclusion criteria for the study described in the methods.<br><br>Individuals in the chronic group (n=22, n=15 males, age range: 22-58yrs) were recruited from the community via the Blusson Spinal Cord Centre (BSCC), Vancouver, BC, Canada from April 2014-September 2016. These individuals were recruited to the study on providing informed consent and meeting the inclusion and exclusion criteria for the study described in the methods. The individuals may be subject to selection and sampling bias as these individuals were part of the local spinal cord community who visit the medical facilities and recreation centre regularly. However, given the sample size of this group, the study includes a higher number of individuals with chronic SCI compared to the wider literature.<br><br>For the penile vibrostimulation studies, data from 10 cervical SCI participants (all male, age range: 22-47yrs) were collected from a secondary analysis of a prospective study.<br><br>Control participants were not compensated as part of this study. Participants with SCI were compensated with a monetary honorarium for their time.
Ethics oversight	All protocols and procedures were compliant with the second Helsinki Declaration, and ethical approval was obtained from the University of British Columbia Clinical Research Ethics Board (H13-03072 and H13-02991).

Note that full information on the approval of the study protocol must also be provided in the manuscript.
